# No Evidence That HIV-1 Subtype C Infection Compromises the Efficacy of Tenofovir-Containing Regimens: Cohort Study in the United Kingdom

**DOI:** 10.1093/infdis/jiw213

**Published:** 2016-05-24

**Authors:** Ellen White, Erasmus Smit, Duncan Churchill, Simon Collins, Clare Booth, Anna Tostevin, Caroline Sabin, Deenan Pillay, David T. Dunn

**Affiliations:** 1 MRC Clinical Trials Unit at UCL; 2 Department of Infection and Population Health; 3 Division of Infection and Immunity, University College London; 4 HIV i-Base; 5 Health Service Laboratories, Royal Free Hospital, London; 6 Public Health England, Birmingham Heartlands Hospital; 7 Brighton and Sussex Hospitals NHS Trust, United Kingdom; 8 Africa Centre for Population Health, University of KwaZulu-Natal, Durban, South Africa

**Keywords:** tenofovir, HIV-1, subtype, virological failure, K65R

## Abstract

Concern has been expressed that tenofovir-containing regimens may have reduced effectiveness in the treatment of human immunodeficiency virus type 1 (HIV-1) subtype C infections because of a propensity for these viruses to develop a key tenofovir-associated resistance mutation. We evaluated whether subtype influenced rates of virological failure in a cohort of 8746 patients from the United Kingdom who received a standard tenofovir-containing first-line regimen and were followed for a median of 3.3 years. In unadjusted analyses, the rate of failure was approximately 2-fold higher among patients infected with subtype C virus as compared to those with subtype B virus (hazard ratio [HR], 1.86; 95% confidence interval [CI], 1.50–2.31; *P* < .001). However, the increased risk was greatly attenuated in analyses adjusting for demographic and clinical factors (adjusted HR, 1.14; 95% CI, .83–1.58; *P* = .41). There were no differences between subtypes C and subtypes non-B and non-C in either univariate or multivariate analysis. These observations imply there is no intrinsic effect of viral subtype on the efficacy of tenofovir-containing regimens.

**(See the editorial commentary by Günthard and Scherrer on pages 1289–91.)**

Human immunodeficiency virus type 1 (HIV-1) subtype C viruses have a predisposition to develop the K65R mutation in reverse transcriptase, an observation first reported in cell culture experiments [1] and subsequently verified in clinical populations [2]. The likely mechanism for this effect is the poly-adenine stretch at codons 63–65 in subtype C viruses, which leads to template pausing at codon 65 [3]. This phenomenon raises serious public health concerns for 2 reasons. First, subtype C is the most common viral subtype worldwide, accounting for around 50% of all infections [4]. Second, tenofovir, which selects for K65R, has been a World Health Organization (WHO)–recommended first-line antiretroviral drug since 2013 and is increasingly being used in all geographical regions [5, 6]. Concern was underlined in a recent article from the TenoRes collaboration, which analyzed tenofovir resistance in patients who received a first-line regimen containing tenofovir plus a cytosine analogue and a non-nucleotide reverse-transcriptase inhibitor, based on combined data from cohorts and clinical trials across 36 countries [7]. Substantial regional variation was found, with an alarmingly high prevalence of tenofovir resistance in sub-Saharan Africa (57%). A smaller South African study that used both Sanger sequencing and more sensitive methods showed that Sanger sequencing frequently misses the K65R mutation [8]. However, these studies only included patients with virological failure and were therefore unable to provide estimates of the risk of virological failure (or tenofovir resistance) among all patients initiating tenofovir-containing regimens.

Data on the relationship between viral subtype and response to antiretroviral therapy are surprisingly limited, although 2 recent studies reported a higher rate of virological failure associated with subtype C infection [9, 10]. Here, we present data from a large cohort study conducted in the United Kingdom, which has a universal public healthcare system and a highly diverse HIV epidemic with a wide representation of viral subtypes [11]. In contrast to previous studies, we have restricted attention to outcomes on standard first-line tenofovir-containing regimens, to maximize relevance to current clinical practice.

## METHODS

The UK Collaborative HIV Cohort Study (available at: http://www.ukchic.org.uk) collates routinely collected data on HIV-infected patients who have attended ≥1 of the collaborating clinics since 1 January 1996. Patients were eligible for the present analysis if they initiated a first-line regimen that contained tenofovir, plus lamivudine or emtricitabine, and either a nonnucleoside reverse transcriptase inhibitor (efavirenz or nevirapine) or a ritonavir-boosted protease inhibitor (lopinavir, atazanavir, or darunavir). In addition, they were required to have sufficient follow-up to potentially meet the criteria for virological failure—this was defined as the first of 2 consecutive viral loads (<6 months apart) of >200 copies/mL after 6 months of antiretroviral therapy. Follow-up was censored at the earlier of the last viral load measurement or the discontinuation of tenofovir, ignoring changes in the prescription of other antiretroviral drugs. Viral subtype was generated from linked pol gene sequences collated by the UK HIV Drug Resistance Database (available at: http://www.hivrdb.org.uk), using the Rega subtyping tool (version 3) [12], and grouped as B, C, or non-B and non-C (hereafter, “non-B/C”). Although almost all non-B/C viruses have the same nucleotide template as subtype B at codons 64–66 in reverse transcriptase [13], we compared them separately against subtype C viruses, rather than combining 2 groups with markedly different demographic characteristics.

Information on viral subtype was available from both resistance tests conducted prior to treatment initiation and tests conducted at virological failure. Conventionally, the latter tests are ignored in analyses of the association between subtype and virological outcomes because they inflate estimates of virological failure rates. In a more efficient approach, we considered all resistance tests and included all patients, even if they had not had a resistance test, using multiple imputation to fill in missing values for subtype. As there were other covariates with incomplete information, we used a technique called chained multiple imputation which fits a series of iterative models appropriate to the type of variable (linear for continuous, logistic for binary, and multinomial logistic regression for categorical). The imputation models included all covariates included in the analysis model (subtype, specific NNRTI/PI, exposure group, ethnicity, baseline viral load, baseline CD4^+^ T-cell count, and date of initiation of antiretroviral therapy), year of diagnosis, and region of birth (countries were grouped into regions with similar subtype distributions [4]). The latter variable is a particularly powerful predictor of viral subtype. Virological outcome, as an indicator variable and cumulative hazard at the time of failure/censoring, was also included in the imputation model to avoid underestimating the association between viral subtype and virological failure [14]. Cox proportional hazards models were used to model the time to virological failure. Continuous variables were fitted using multivariable fractional polynomials, first using the complete case data to define the power indices. Multivariable transformations were included as just another variable in the imputation models [15]. We performed 10 imputations and combined results using Rubin's rule. Unadjusted (Kaplan–Meier) and adjusted (from Cox model) survivor functions were derived by averaging survival at each time point over the imputed data sets.

Tenofovir resistance was defined as the presence of a K65R or K70E mutation in the reverse transcriptase gene [16]. We compared the frequency of tenofovir resistance between viral subtypes on the basis of resistance tests conducted between 30 days before and 90 days after the date of virological failure as defined above. Fisher's exact tests were used to assess statistical significance.

The UK Collaborative HIV Cohort Study and the UK HIV Drug Resistance Database have separate multicentre research ethics approvals, which waived the requirement for individual patient consent. All analyses were conducted using Stata 13.1.

## RESULTS

A total of 8746 patients were included in the analysis, of whom 6149 (70.3%) had a determined subtype: 4123 had subtype B, 823 had subtype C, and 1203 had non-B/C subtypes. The most common non-B/C subtypes were A (n = 272), CRF02_AG (n = 267), G (n = 118), CRF01_AE (n = 114), other CRFs (n = 90), and D (n = 80). Baseline characteristics are shown in Table 1. Patients infected with subtype B viruses were mainly white (82.7%) and men who have sex with men (MSM; 85.0%), whereas the subtype C group was mainly black (70.2%) and heterosexual (79.2%). The non-B/C group was more heterogeneous, with 34.9% white, 53.0% black, 25.6% MSM, and 63.1% heterosexual. Overall, 74.4% of first-line regimens included a NNRTI (mostly efavirenz), while 25.6% included a boosted PI. First-line regimens were broadly comparable across the different viral subtype groups, although there was proportionately greater use of efavirenz for subtype B and proportionately greater use of lopinavir for subtype C.

**Table 1.** Baseline Characteristics, by Human Immunodeficiency Virus (HIV) Subtype

Characteristic	Subtype			
	B (n = 4123)	C (n = 823)	Non-B/C (n = 1203)	Missing (n = 2597)
Year at ART initiation	2008 (2007–2010)	2009 (2007–2010)	2009 (2008–2010)	2008 (2006–2010)
Year at HIV diagnosis	2005 (2003–2008)	2007 (2004–2009)	2007 (2005–2009)	2005 (2003–2008)
Age at ART initiation, year	38 (32–44)	38 (32–44)	38 (31–45)	38 (32–45)
Viral load at ART initiation, copies/mL	68 600 (16 400–194 800)	49 600 (9500–194 100)	61 200 (10 400–227 500)	25 300 (200–120 000)
CD4^+^ T-cell count at ART initiation, cells/mm^3^	258 (175–337)	204 (100–297)	214 (100–301)	244 (142–361)
Ethnicity				
White	3409 (82.7)	159 (19.3)	420 (34.9)	1305 (50.3)
Black	248 (6.0)	578 (70.2)	637 (53.0)	995 (38.3)
Asian	129 (3.1)	32 (3.9)	53 (4.4)	99 (3.8)
Other	286 (6.9)	43 (5.2)	75 (6.2)	145 (5.6)
Unknown	51 (1.2)	11 (1.3)	18 (1.5)	53 (2.0)
Exposure group				
MSM	3503 (85.0)	93 (11.3)	308 (25.6)	1152 (44.4)
Heterosexual sex, males	167 (4.1)	265 (32.2)	341 (28.4)	532 (20.5)
Heterosexual sex, females	113 (2.7)	387 (47.0)	418 (34.8)	659 (25.4)
Other	298 (7.2)	56 (6.8)	106 (8.8)	178 (6.9)
Unknown	42 (1.0)	22 (2.7)	30 (2.5)	76 (2.9)
First-line regimen				
TDF + 3TC/FTC + EFV	2893 (70.2)	546 (66.3)	787 (65.4)	1752 (67.5)
TDF + 3TC/FTC + NVP	158 (3.8)	46 (5.6)	84 (7.0)	238 (9.2)
TDF + 3TC/FTC + ATV/r	428 (10.4)	78 (9.5)	132 (11.0)	231 (8.9)
TDF + 3TC/FTC + DRV/r	280 (6.8)	51 (6.2)	98 (8.2)	156 (6.0)
TDF + 3TC/FTC + LPV/r	364 (8.8)	102 (12.4)	102 (8.5)	220 (8.5)

Data are no. (%) of subjects or median value (interquartile range).

Abbreviations: 3TC, lamivudine; ART, antiretroviral therapy; ATV/r, atazanavir/ritonavir; DRV/r, darunavir/ritonavir; EFV, efavirenz; FTC, emtricitabine; LPV/r, lopinavir/ritonavir; MSM, men who have sex with men; NVP, nevirapine; TDF, tenofovir disoproxil fumarate.

The 2597 patients without a resistance test were assigned, averaging over imputations, to subtype B (n = 1342), subtype C (n = 632), and non-B/C subtypes (n = 623). This resulted in total (average) sample sizes of 5465, 1455, and 1826, respectively. The proportionate increase in sample size, relative to those with known subtype, was greatest for subtype C (77%), lowest for subtype B (33%), and intermediate for subtype non-B/C (52%), reflecting variation in the availability of resistance tests by demographic and clinical characteristics associated with viral subtype.

**Figure 1.**
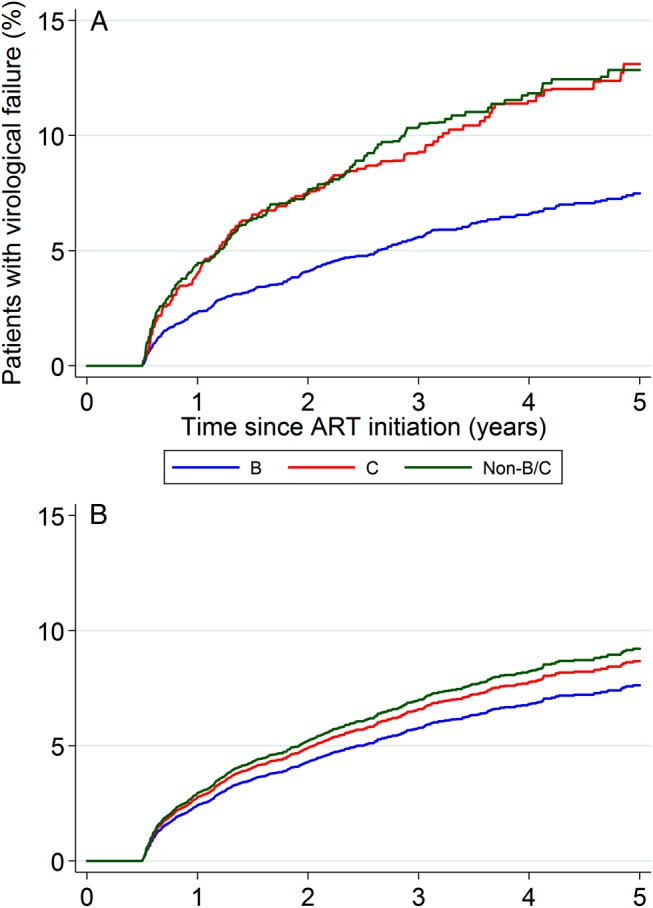
*A*, Unadjusted Kaplan–Meier analysis. *B*, Estimated survivor function from Cox modeling. Abbreviation: ART, antiretroviral therapy.

Patients were followed up for a median of 3.3 years (interquartile range [IQR], 2.0–4.9 years). On average, 309 (5.7%) of subtype B–infected patients, 142 (9.8%) of subtype C–infected patients, and 173 (9.5%) of non-B/C subtype–infected patients experienced virological failure (Figure 1*A* and Table 2). In unadjusted analyses, the rate of failure was approximately 2-fold higher among patients infected with subtype C virus as compared to those infected with subtype B virus (ratio of the hazard for subtype B infection vs that for subtype C infection, 0.54; 95% confidence interval [CI], .43–.67; *P* < .001), whereas there was no difference compared with subtype non-B/C (hazard ratio [HR], 1.00; 95% CI, .77–1.31; *P* = .98). The estimated cumulative risk of virological failure at 5 years was 7.5% (95% CI, 6.6%–8.5%) for subtype B, 13.1% (95% CI, 10.9%–15.7%) for subtype C, and 12.8% (95% CI, 10.9%–15.1%) for subtype non-B/C (Figure 1*A*). Adjustment for demographic and clinical factors had a marked effect on the comparison of subtypes B and C (Figure 1*B* and Table 2), with the virological failure rate estimated to be only 13% lower for subtype B, which was not statistically significant (HR, 0.87; 95% CI, .63–1.21; *P* = .41). The comparison between subtype non-B/C and subtype C remained relatively unchanged (HR, 1.06; 95% CI, .81–1.40; *P* = .65). Adjusted Kaplan–Meier curves of the time to virological failure confirmed the lack of association with viral subtype, after accounting for potential confounders (Figure 1*B*). Similar relationships between subtypes were observed in a sensitivity analysis of the complete case data (Supplementary Table 1).

**Table 2.** Predictors of Virological Failure (VF)

Predictor	Total Subjects, No.	Subjects With VF, No. (%)	HR (95% CI)	Adjusted HR^a^ (95% CI)	*P* Value
HIV subtype					
B	5465	309 (5.7)	0.54 (.43–.67)	0.87 (.63–1.21)	.41^b^
C	1455	142 (9.8)	1.00	1.00	
Non-B/C	1826	173 (9.5)	1.00 (.77–1.31)	1.06 (.81–1.40)	.65^b^
First-line regimen					<.001
TDF + 3TC/FTC + EFV	5978	345 (5.8)	1.00	1.00	
TDF + 3TC/FTC + NVP	526	46 (8.8)	1.43 (1.05–1.94)	1.38 (1.01–1.89)	
TDF + 3TC/FTC + ATV/r	869	97 (11.2)	2.07 (1.65–2.59)	2.07 (1.64–2.59)	
TDF + 3TC/FTC + DRV/r	585	50 (8.6)	2.13 (1.58–2.87)	2.05 (1.50–2.80)	
TDF + 3TC/FTC + LPV/r	788	86 (10.9)	1.73 (1.37–2.20)	1.48 (1.16–1.89)	
Exposure group					<.001
MSM	5127	253 (4.9)	1.00	1.00	
Heterosexual sex, males	1342	138 (10.3)	2.26 (1.83–2.78)	1.63 (1.21–2.21)	
Heterosexual sex, females	1623	158 (9.7)	2.14 (1.75–2.61)	1.47 (1.07–2.00)	
Other	653	75 (11.5)	2.60 (2.00–3.37)	2.48 (1.88–3.27)	
Ethnicity					.04
White	5367	314 (5.9)	1.00	1.00	
Black	2499	262 (10.5)	1.91 (1.62–2.25)	1.33 (1.03–1.71)	
Asian	321	16 (5.0)	0.89 (.54–1.48)	0.79 (.48–1.32)	
Other	559	32 (5.7)	0.97 (.68–1.40)	0.88 (.61–1.28)	
Baseline HIV RNA level, copies/mL^c^					<.001
5000	…	…	1.00	1.00	
10 000	…	…	1.04 (1.02–1.07)	1.05 (1.02–1.07)	
50 000	…	…	1.23 (1.14–1.33)	1.23 (1.14–1.34)	
100 000	…	…	1.36 (1.23–1.51)	1.35 (1.21–1.51)	
250 000	…	…	1.59 (1.37–1.86)	1.55 (1.32–1.83)	
Baseline CD4^+^ T-cell count, cells/mm^3c^					.02
100	…	…	1.00	1.00	
200	…	…	0.83 (.79–.87)	0.93 (.88–.99)	
300	…	…	0.74 (.69–.80)	0.90 (.82–.98)	
400	…	…	0.69 (.63–.76)	0.87 (.78–.98)	
500	…	…	0.65 (.58–.72)	0.85 (.75–.97)	
Date of ART initiation (per calendar year)	…	…	0.97 (.94–1.01)	0.97 (.93–1.01)	.17

Data are averages over imputed data sets.

Abbreviations: 3TC, lamivudine; ART, antiretroviral therapy; ATV/r, atazanavir/ritonavir; CI, confidence interval; DRV/r, darunavir/ritonavir; EFV, efavirenz; FTC, emtricitabine; HIV, human immunodeficiency virus; HR, hazard ratio; LPV/r, lopinavir/ritonavir; MSM, men who have sex with men; NVP, nevirapine; TDF, tenofovir disoproxil fumarate.

^a^ Adjusted for all variables in table.

^b^ By individual Wald tests.

^c^ HRs are presented at selected values as fitted as nonlinear, continuous relationship. Data are also shown in Supplementary Figure 1.

We explored the reasons for the change in the estimated effect of subtype between unadjusted and adjusted analyses by fitting a series of models containing various combinations of the various factors. The most influential factors were exposure group (lower rate of virological failure among MSM) and ethnicity (lower rate of virological failure among white and Asian patients). Antiretroviral regimens that included an NNRTI (particularly efavirenz) were significantly more durable than regimens that included a boosted PI, although this may due to channeling bias, rather than an intrinsic pharmacological effect [17]. Nonlinear effects were observed for baseline CD4^+^ T-cell count and baseline viral load. Virological failure was much more frequent for a baseline CD4^+^ T-cell count of <100 cells/mm^3^ and much less frequent for a baseline viral load of <100 000 copies/mL. There was a nonsignificant trend toward a lower rate of virological failure if antiretroviral therapy was initiated in more-recent years (Table 2, Supplementary Figure 1).

Genotypic resistance tests were available for 260 of 624 patients (41.7%) who experienced virological failure (Table 3). Tenofovir-associated resistance mutations, predominantly K65R, were observed significantly more frequently in subtype C viruses (22.7%) than in subtype B virus (6.1%) or non-B/C viruses (8.1%; *P* = .003).

**Table 3.** Tenofovir Resistance at Virological Failure (VF), by Human Immunodeficiency Virus Subtype

Variable	Subtype			*P* Value^a^
	B	C	Non-B/C	
Experienced VF	309	142	173	
Underwent resistance testing^b^	132 (42.7)	66 (46.5)	62 (35.8)	
Major TDF mutation detected^c^	8 (6.1)	15 (22.7)	5 (8.1)	.003
K65R	6 (4.6)	13 (19.7)	4 (6.5)	.003
K70E	2 (1.5)	2 (3.0)	1 (1.6)	.84

Data are no. or no. (%) of subjects.

Abbreviation: TDF, tenofovir disoproxil fumarate.

^a^ By the χ^2^ test with 2 degrees of freedom.

^b^ Conducted between 30 days before and 90 days after failure.

^c^ K65R or K70E mutation.

## DISCUSSION

In the present study patients infected with HIV-1 subtype C viruses who were receiving a first-line tenofovir-containing regimen experienced a higher rate of virological failure than patients infected with subtype B viruses. However, this effect was almost entirely explained by differences between the groups in demographic and clinical characteristics, particularly exposure group and ethnicity, possibly related to differential nonadherence [18]. Also, the distribution of time to virological failure among patients infected with subtype non-B/C viruses, which overwhelmingly share the same nucleotide template as subtype B at the codons critical for the development of the primary tenofovir mutation (K65R), closely mirrored the distribution for patients infected with subtype C viruses. These observations imply that there is no intrinsic effect of viral subtype on the efficacy of tenofovir-containing regimens, and they specifically alleviate concerns that efficacy would be compromised for subtype C infections [19]. This is a reassuring finding in light of the rapid worldwide expansion in the prescribing of tenofovir and supports WHO recommendations that this is an appropriate first-line drug, even in geographical regions where subtype C HIV-1 infection is endemic.

Although several studies have previously examined the association between viral subtype and response to therapy, all included a spectrum of different NRTI backbones, which could have masked a specific effect of tenofovir. A further general limitation has been the combined analysis of subtype C infections with other subtype non-B infections because of limited numbers. A meta-analysis of European cohort studies reported almost identical virological outcomes among approximately 7000 patients infected with subtype B virus and 700 patients infected with subtype C virus, but patients were followed for a maximum of only 16 months, and all 3-drug regimens were considered [20]. A French study of patients with primary HIV infection similarly found that immunological and virological responses were unaffected by viral subtype, but this analysis included only 12 subjects with subtype C infection [21]. In an analysis limited to white patients, the Swiss HIV Cohort Study found superior virological outcomes in subjects infected with a non-B subtype, but only 18% of patients were receiving a tenofovir-containing regimen, and only 13% of the non-B infections were subtype C [22]. A recent article from Sweden reported an increased rate of virological failure in patients with subtype C infection but did not report the NRTIs that were prescribed (their primary interest was the interaction between subtype and NNRTI-based vs PI-based regimens) [9]. A similar finding was reported in a nested case-cohort study of AIDS Clinical Trials Group A5175, which randomly allocated patients to receive one of 3 first-line regimens [10]. However, only one of these regimens included tenofovir (combined with efavirenz and emtricitabine), and there were too few end points to examine whether the effect of subtype depended upon the specific first-line regimen [19]. It is also noted that this was a multinational study—with most subtype C–infected patients presumably enrolled from sites in southern Africa—and that any comparison of subtypes is conflated with geographical variation. Although procedures are standardized in randomized controlled trials, some local variation in clinical care and patient behavioral characteristics inevitably remain.

Our study has several strengths. First, it is the only study that has exclusively examined tenofovir-containing first-line regimens, which makes it highly relevant to current practice. Second, the data were derived from a single national health system that offers near uniform care and thus avoids confounding biases inherent in multinational studies. Third, our estimates of effect are relatively precise because of the large absolute number of virological failure end points and the presence of all major viral subtypes in the United Kingdom. Finally, the diversification of the epidemic, particularly the spread of non-B viruses in the MSM population [11], has allowed us to segregate the effect of viral subtype per se from the confounding effects of exposure group and ethnicity.

Further research is required to reconcile the paradox of the absence of a subtype effect on virological response with the observation that subtype C viruses are more likely to express the K65R mutation in viremic patients [2], which was confirmed in the present analysis. This is a complex problem since the rate of generation of new mutations and outgrowth of resistant strains is a function of the level of viral replication while the level of viral replication is a function of the degree of viral susceptibility to drugs in the current regimen. We note that K65R confers only partial resistance to tenofovir; for example, of 66 subtype B isolates with K65R in the Stanford HIV Drug Resistance Database, the median phenotypic fold-resistance (by the Phenosense assay) was only 1.7 [23]. In addition, analyses relating short-term virological resistance to predicted phenotype in patients switching therapy suggest that tenofovir retains appreciable antiviral activity if resistance is less than approximately 2-fold [24]. There may therefore be an extended interval between the emergence of the K65R mutation and the manifestation of virological failure. The long-term follow-up in our study is important in this regard. The overall cumulative rate of virological failure of only 9% at 5 years attests to the durability of first-line tenofovir-containing regimens, regardless of viral subtype.

## MEMBERS OF THE STUDY GROUP

Collaborators in the UK CHIC study are listed in the Supplementary Material. The UK HIV Drug Resistance Database steering committee comprises the following individuals: Celia Aitken (Gartnavel General Hospital, Glasgow); David Asboe, Anton Pozniak (Chelsea and Westminster Hospital, London); Patricia Cane (Public Health England, Porton Down); David Chadwick (South Tees Hospitals NHS Trust, Middlesbrough); Duncan Churchill (Brighton and Sussex University Hospitals NHS Trust); Duncan Clark (St Bartholomew's and The London NHS Trust); Simon Collins (HIV i-Base, London); Valerie Delpech (Centre for Infections, Public Health England); Samuel Douthwaite (Guy's and St. Thomas' NHS Foundation Trust, London); David Dunn, Esther Fearnhill, Kholoud Porter, Anna Tostevin, Ellen White (MRC Clinical Trials Unit at UCL, London); Christophe Fraser (Imperial College London); Anna Maria Geretti (Institute of Infection and Global Health, University of Liverpool); Antony Hale (Leeds Teaching Hospitals NHS Trust); Stéphane Hué (London School of Hygiene and Tropical Medicine, London); Steve Kaye (Imperial College, London); Paul Kellam (Wellcome Trust Sanger Institute and University College London Medical School); Linda Lazarus (Expert Advisory Group on AIDS Secretariat, Public Health England); Andrew Leigh-Brown (University of Edinburgh); Tamyo Mbisa (Virus Reference Department, Public Health England); Nicola Mackie (Imperial NHS Trust, London); Samuel Moses (King's College Hospital, London); Chloe Orkin (St. Bartholomew's Hospital, London); Eleni Nastouli, Deenan Pillay, Andrew Phillips, Caroline Sabin (University College London Medical School, London); Erasmus Smit (Public Health England, Birmingham Heartlands Hospital); Kate Templeton (Royal Infirmary of Edinburgh); Peter Tilston (Manchester Royal Infirmary); Ian Williams (Mortimer Market Centre, London); Hongyi Zhang (Addenbrooke's Hospital, Cambridge).

The coordinating center (and affiliated individuals) is the MRC Clinical Trials Unit at UCL (David Dunn, Keith Fairbrother, Esther Fearnhill, Kholoud Porter, Anna Tostevin, Ellen White).

The following centers (and affiliated individuals) contribute data: Clinical Microbiology and Public Health Laboratory, Addenbrooke's Hospital, Cambridge (Jane Greatorex); Guy's and St. Thomas' NHS Foundation Trust, London (Siobhan O'Shea, Jane Mullen); PHE – Public Health Laboratory, Birmingham Heartlands Hospital, Birmingham (Erasmus Smit); PHE – Virus Reference Department, London (Tamyo Mbisa); Imperial College Health NHS Trust, London (Alison Cox); King's College Hospital, London (Richard Tandy); Medical Microbiology Laboratory, Leeds Teaching Hospitals NHS Trust (Tracy Fawcett); Specialist Virology Centre, Liverpool (Mark Hopkins, Lynne Ashton); Department of Clinical Virology, Manchester Royal Infirmary, Manchester (Peter Tilston); Department of Virology, Royal Free Hospital, London (Clare Booth, Ana Garcia-Diaz); Edinburgh Specialist Virology Centre, Royal Infirmary of Edinburgh (Jill Shepherd); Department of Infection and Tropical Medicine, Royal Victoria Infirmary, Newcastle (Matthias L Schmid, Brendan Payne); South Tees Hospitals NHS Trust, Middlesbrough (David Chadwick); Department of Virology, St Bartholomew's and The London NHS Trust (Duncan Clark, Jonathan Hubb); Molecular Diagnostic Unit, Imperial College, London (Steve Kaye); University College London Hospitals (Stuart Kirk); West of Scotland Specialist Virology Laboratory, Gartnavel, Glasgow (Rory Gunson, Amanda Bradley-Stewart, Celia Aitken).

## Supplementary Data

Supplementary materials are available at http://jid.oxfordjournals.org. Consisting of data provided by the author to benefit the reader, the posted materials are not copyedited and are the sole responsibility of the author, so questions or comments should be addressed to the author.

Supplementary Data
