# Supplementary material for: No Evidence That HIV-1 Subtype C Infection Compromises the Efficacy of Tenofovir-Containing Regimens: Cohort Study in the United Kingdom
Source: J Infect Dis. 2016 May 24;214(9):1302–8. doi: 10.1093/infdis/jiw213 (PMC5079361; doi:10.1093/infdis/jiw213)
Supplement: Supplementary Data [file supp_214_9_1302__index.html]

No Evidence That HIV-1 Subtype C Infection Compromises the Efficacy of Tenofovir-Containing Regimens: Cohort Study in the United Kingdom — No Evidence That HIV-1 Subtype C Infection Compromises the Efficacy of Tenofovir-Containing Regimens: Cohort Study in the United Kingdom — Supplementary Data 

# No Evidence That HIV-1 Subtype C Infection Compromises the Efficacy of Tenofovir-Containing Regimens: Cohort Study in the United Kingdom

## Supplementary Data

Supplementary Data

- Supplementary data - docx file
- Supplementary UKCHICcollaborators - doc file
