# Supplementary material for: No Evidence That HIV-1 Subtype C Infection Compromises the Efficacy of Tenofovir-Containing Regimens: Cohort Study in the United Kingdom
Source: J Infect Dis. 2016 May 24;214(9):1302–8. doi: 10.1093/infdis/jiw213 (PMC5079361; doi:10.1093/infdis/jiw213)
Supplement: Supplementary Data [file supp_jiw213_jiw213supp_data.docx]

**Supplementary Tables and Figures**

**Supplementary Table 1: Predictors of virological failure using a sensitivity analysis of data for patients with complete covariates**

|  | **Total** | **VF**  **N (%)** | **HR** | **aHR*** | **95% CI** | **p-value** |
| --- | --- | --- | --- | --- | --- | --- |
| **Subtype** |  |  |  |  |  |  |
| B | 3765 | 194 (5.2) | 0.46 | 0.96 | 0.65-1.40 | 0.81** |
| C | 684 | 71 (10.4) | 1.00 | 1.00 | --- |  |
| Non-B Non-C | 1023 | 92 (9.0) | 0.90 | 0.98 | 0.72-1.34 | 0.90** |
| **First -line regimen** |  |  |  |  |  | <0.001 |
| TDF + 3TC/FTC + EFV | 3804 | 206 (5.4) | 1.00 | 1.00 | --- |  |
| TDF + 3TC/FTC + NVR | 248 | 27 (10.9) | 1.93 | 1.79 | 1.19-2.69 |  |
| TDF + 3TC/FTC + ATA/r | 555 | 55 (9.9) | 1.93 | 1.86 | 1.38-2.51 |  |
| TDF + 3TC/FTC + DRV/r | 375 | 24 (6.4) | 1.63 | 1.49 | 0.96-2.31 |  |
| TDF + 3TC/FTC + LPV/r | 490 | 45 (9.2) | 1.54 | 1.26 | 0.90-1.76 |  |
| **Exposure group** |  |  |  |  |  | <0.001 |
| MSM | 3618 | 157 (4.3) | 1.00 | 1.00 | --- |  |
| MSF | 657 | 73 (11.1) | 2.81 | 2.18 | 1.50-3.19 |  |
| FSM | 792 | 82 (10.4) | 2.67 | 1.91 | 1.28-2.84 |  |
| Other | 405 | 45 (11.1) | 2.89 | 2.92 | 2.06-4.14 |  |
| **Ethnicity** |  |  |  |  |  | 0.04 |
| White | 3661 | 195 (5.3) | 1.00 | 1.00 | --- |  |
| Black | 1247 | 138 (11.1) | 2.25 | 1.36 | 0.98-1.89 |  |
| Asian | 189 | 7 (3.7) | 0.70 | 0.58 | 0.27-1.25 |  |
| Other | 375 | 17 (4.5) | 0.84 | 0.76 | 0.46-1.25 |  |
| **Baseline HIV RNA (copies/ml)***** | |  |  |  |  | <0.001 |
| 5,000 |  |  | 1.00 | 1.00 | --- |  |
| 10,000 |  |  | 1.03 | 1.04 | 1.00-1.07 |  |
| 50,000 |  |  | 1.20 | 1.22 | 1.10-1.36 |  |
| 100,000 |  |  | 1.35 | 1.36 | 1.19-1.57 |  |
| 250,000 |  |  | 1.62 | 1.63 | 1.34-1.98 |  |
| **Baseline CD4 count (cells/mm^3^)***** | |  |  |  |  | 0.21 |
| 100 |  |  | 1.00 | 1.00 | --- |  |
| 200 |  |  | 0.82 | 0.95 | 0.89-1.03 |  |
| 300 |  |  | 0.74 | 0.93 | 0.83-1.04 |  |
| 400 |  |  | 0.68 | 0.91 | 0.79-1.06 |  |
| 500 |  |  | 0.64 | 0.90 | 0.76-1.06 |  |
| **Date of ART initiation (per calendar year)** | | |  |  |  |  |
|  |  |  | 0.98 | 0.98 | 0.92-1.03 | 0.42 |
| *Adjusted for all variables in table  **P-values from individual Wald tests  ***Hazard ratios presented at selected values as fitted as non-linear, continuous relationship.  Values shown are averages over imputed datasets | | | | | | |

**Supplementary Figure 1: Predicted hazard ratios from adjusted Cox model**

**
**

**Supplementary Figure 1 Legend:** (A) Year of ART initiation, (B) pre-ART viral load, (C) pre-ART CD4 count.

**Supplementary Table 2: Patterns of complete data for analysis covariates**

|  |  | **Subtype** | **Exposure** | **Ethnicity** | **Baseline RNA** | **Baseline CD4** |
| --- | --- | --- | --- | --- | --- | --- |
| **Number** | **%** |  |  |  |  |  |
| 5693 | 65 |  |  |  |  |  |
| 1714 | 20 |  |  |  |  |  |
| 522 | 6 |  |  |  |  |  |
| 171 | 2 |  |  |  |  |  |
| 156 | 2 |  |  |  |  |  |
| 490 | 5 | Other patterns | | | | |
| *Shaded boxes show patterns of complete data for analysis model. First-line regimen and year of ART available for all individuals. | | | | | | |
