# Supplementary material for: No Evidence That HIV-1 Subtype C Infection Compromises the Efficacy of Tenofovir-Containing Regimens: Cohort Study in the United Kingdom
Source: J Infect Dis. 2016 May 24;214(9):1302–8. doi: 10.1093/infdis/jiw213 (PMC5079361; doi:10.1093/infdis/jiw213)
Supplement: Supplementary Data [file supp_jiw213_jiw213supp_UKCHICcollaborators.doc]

# **UK CHIC collaborators**

***Steering Committee:*** Jonathan Ainsworth, Sris Allan, Jane Anderson, Abdel Babiker, David Chadwick, Valerie Delpech, David Dunn, Martin Fisher, Brian Gazzard, Richard Gilson, Mark Gompels, Phillip Hay, Teresa Hill, Margaret Johnson, Sophie Jose, Stephen Kegg, Clifford Leen, Mark Nelson, Chloe Orkin, Adrian Palfreeman, Andrew Phillips, Deenan Pillay, Frank Post, Jillian Pritchard, Caroline Sabin, Memory Sachikonye, Achim Schwenk, Anjum Tariq, John Walsh.

***Central Co-ordination****: University College London* (Teresa Hill, Sophie Jose, Andrew Phillips, Caroline Sabin, Alicia Thornton); *Medical Research Council Clinical Trials Unit at UCL (MRC CTU at UCL), London* (David Dunn, Adam Glabay).

***Participating Centres:*** *Barts Health NHS Trust, London* (Chloe Orkin, Janet Lynch, James Hand, Carl de Souza); *Brighton and Sussex University Hospitals NHS Trust* (Martin Fisher, Nicky Perry, Stuart Tilbury, Elaney Youssef, Duncan Churchill); *Chelsea and Westminster Hospital NHS Foundation Trust, London* (Brian Gazzard, Mark Nelson, Rhiannon Everett, David Asboe, Sundhiya Mandalia); *Public Health England, London* (Valerie Delpech); *Homerton University Hospital NHS Trust, London* (Jane Anderson, Sajid Munshi); *King’s College Hospital NHS Foundation Trust, London* (Frank Post, Ade Adefisan, Chris Taylor, Zachary Gleisner, Fowzia Ibrahim, Lucy Campbell); *Medical Research Council Clinical Trials Unit (MRC CTU), London* (Abdel Babiker, David Dunn, Adam Glabay); Middlesbrough, South Tees Hospitals NHS Foundation Trust, (David Chadwick, Kirsty Baillie); *Mortimer Market Centre, University College London* (Richard Gilson, Nataliya Brima, Ian Williams); *North Middlesex University Hospital NHS Trust, London* (Jonathan Ainsworth, Achim Schwenk, Sheila Miller, Chris Wood); *Royal Free NHS Foundation Trust/University College London* (Margaret Johnson, Mike Youle, Fiona Lampe, Colette Smith, Rob Tsintas, Clinton Chaloner, Samantha Hutchinson, Caroline Sabin, Andrew Phillips Teresa Hill, Sophie Jose, Alicia Thornton, Susie Huntington); *Imperial College Healthcare NHS Trust, London* (John Walsh, Nicky Mackie, Alan Winston, Jonathan Weber, Farhan Ramzan, Mark Carder); *The Lothian University Hospitals NHS Trust, Edinburgh* (Clifford Leen, Alan Wilson, Sheila Morris); *North Bristol NHS Trust* (Mark Gompels, Sue Allan); *Leicester,* *University Hospitals of Leicester NHS Trust* (Adrian Palfreeman, Khurram Memon, Adam Lewszuk); *Woolwich,* *Lewisham and Greenwich NHS Trust* (Stephen Kegg, Akin Faleye, Dr Mitchell, Dr Hunter), *UK Community Advisory Board* (Memory Sachikonye); *St. George’s Healthcare NHS Trust* (Phillip Hay, Mandip Dhillon, Christian Kemble); *York Teaching Hospital NHS Foundation Trust* (Fabiola Martin, Sarah Russell-Sharpe, Janet Gravely); *Coventry, University Hospitals Coventry and Warwickshire NHS Trust* (Sris Allan, Andrew Harte, Stephen Clay); *Wolverhampton, The Royal Wolverhampton Hospitals NHS Trust* (Anjum Tariq, Hazel Spencer, Ron Jones); *Chertsey, Ashford and St.Peter’s Hospitals NHS Foundation Trust* (Jillian Pritchard, Shirley Cumming, Claire Atkinson).

Table of SC members + affiliations

| Sris Allan | University Hospitals Coventry and Warwickshire NHS Trust |
| --- | --- |
| Jonathan Ainsworth | North Middlesex University Hospital NHS Trust, London |
| Jane Anderson | Homerton University Hospital NHS Trust, London |
| Abdel Babiker | MRC Clinical Trials Unit, London |
| Valerie Delpech | Public Health England (PHE), London |
| David Dunn | MRC Clinical Trials Unit, London |
| Frank Post | King’s College Hospital NHS Foundation Trust, London |
| Martin Fisher | Brighton and Sussex University Hospitals NHS Trust |
| Brian Gazzard | Chelsea and Westminster Hospital NHS Foundation Trust, London |
| Richard Gilson | Mortimer Market Centre, Central and North West London NHS Foundation Trust |
| Mark Gompels | North Bristol NHS Trust |
| Phillip Hay | St George’s Healthcare NHS Trust |
| Teresa Hill | University College London |
| Margaret Johnson | Royal Free Foundation NHS Trust, London |
| Sophie Jose | University College London |
| Stephen Kegg | Lewisham and Greenwich NHS Trust, London |
| Clifford Leen | The Lothian University Hospitals NHS Trust |
| Mark Nelson | Chelsea and Westminster Hospital NHS Trust, London |
| Fabiola Martin | York Teaching Hospital NHS Foundation Trust |
| Chloe Orkin | Barts Health NHS Trust, London |
| Andrew Phillips | University College London |
| Deenan Pillay | University College London |
| Jillian Pritchard | Ashford and St. Peter’s Hospitals NHS Foundation Trust |
| Caroline Sabin | University College London |
| Memory Sachikonye | UK Community Advisory Board |
| Achim Schwenk | North Middlesex University Hospital NHS Trust, London |
| Anjum Tariq | The Royal Wolverhampton Hospitals NHS Trust |
| John Walsh/Nicky Mackie | Imperial College Healthcare NHS Trust, London |
| Adrian Palfreeman | University Hospitals of Leicester NHS Trust |
